# SMAS repositioning technique utilizing cog thread: Anatomical perspectives

**DOI:** 10.1111/srt.13650

**Published:** 2024-03-18

**Authors:** Gi‐Woong Hong, Soo‐Bin Kim, Soo Yeon Park, Jovian Wan, Kyu‐Ho Yi

**Affiliations:** ^1^ Samskin Plastic Surgery Clinic Seoul South Korea; ^2^ Division in Anatomy and Developmental Biology Department of Oral Biology Human Identification Research Institute BK21 FOUR Project Yonsei University College of Dentistry Seoul South Korea; ^3^ Made‐Young Plastic Surgery Clinic Seoul South Korea; ^4^ Asia Pacific Aesthetic Academy Hong Kong Hong Kong; ^5^ Maylin Clinic (Apgujeong) Seoul South Korea

**Keywords:** facial anatomy, facial retaining ligament, SMAS repositioning, thread lifting

## Abstract

**Introduction:**

Face‐lifting surgeries were once common among individuals over 60 years old due to skin laxity, but recent trends favor thread lifting in this age group. Understanding dynamic changes in facial anatomy during postural shifts is essential.

**Method:**

Fresh cadaver studies have demonstrated the passage of threads through the superficial musculoaponeurotic system (SMAS) layer, confirming the efficacy of the technique. Proper insertion depth targeting SMAS repositioning, rather than superficial skin layers, is crucial.

**Result:**

The natural movement of tissues secured by thread (N‐Cog and N‐Fix, N‐Finders Inc., Korea) insertion results in lifting effects. However, complications may arise if threads affect deeper facial muscles, leading to discomfort. Fibrous septa play a significant role in guiding thread placement, with different densities influencing thread maneuverability and tissue response during lifting.

**Conclusion:**

Procedures targeting SMAS repositioning using threads aim to maintain the new position of relocated tissues. Understanding structural variations in facial regions informs thread selection and placement. Aligning threads with tissue movement and the intended SMAS layer positioning is vital to prevent complications. Balancing thread insertion depth and tissue traction is critical for successful outcomes. Modern thread lifting techniques prioritize SMAS repositioning, enhancing lifting effects while ensuring procedure safety and efficacy.

## 1 INTRODUCTION

As individuals enter their middle to late years, typically surpassing the age of 60, wrinkles tend to deepen, and skin sagging becomes more pronounced. Consequently, there was a historical necessity for face‐lifting surgeries, involving the pulling and excision of excess skin, owing to the increased laxity of the skin and underlying tissues.^1^ Formerly, it was widely believed that achieving effective results solely through lifting techniques employing threads or sutures was challenging in this age group. However, in recent years, there has been a shift toward performing thread‐lifting procedures in individuals within this age bracket. The commencement of this practice stemmed from a fundamental change in the conceptualization of thread‐lifting techniques.^2^

Previously, the conventional approach involved a straightforward upward traction of the lax skin and subcutaneous tissues.^3^, ^4^, ^5^ Presently, the mindset has evolved to a technique where, when patients are placed in a reclined position, subtly lifting the chin or slightly lowering the head results in the migration of lax tissues of the midface and lower face toward the firmer structures around the head, ears, and jawline. The primary objective of thread lifting is now perceived as securing these migrated connective tissues to the firm underlying structures to prevent them from reverting to their original positions.^6^, ^7^, ^8^

## 2 SMAS REPOSITIONING TECHNIQUE

**FIGURE 1:**
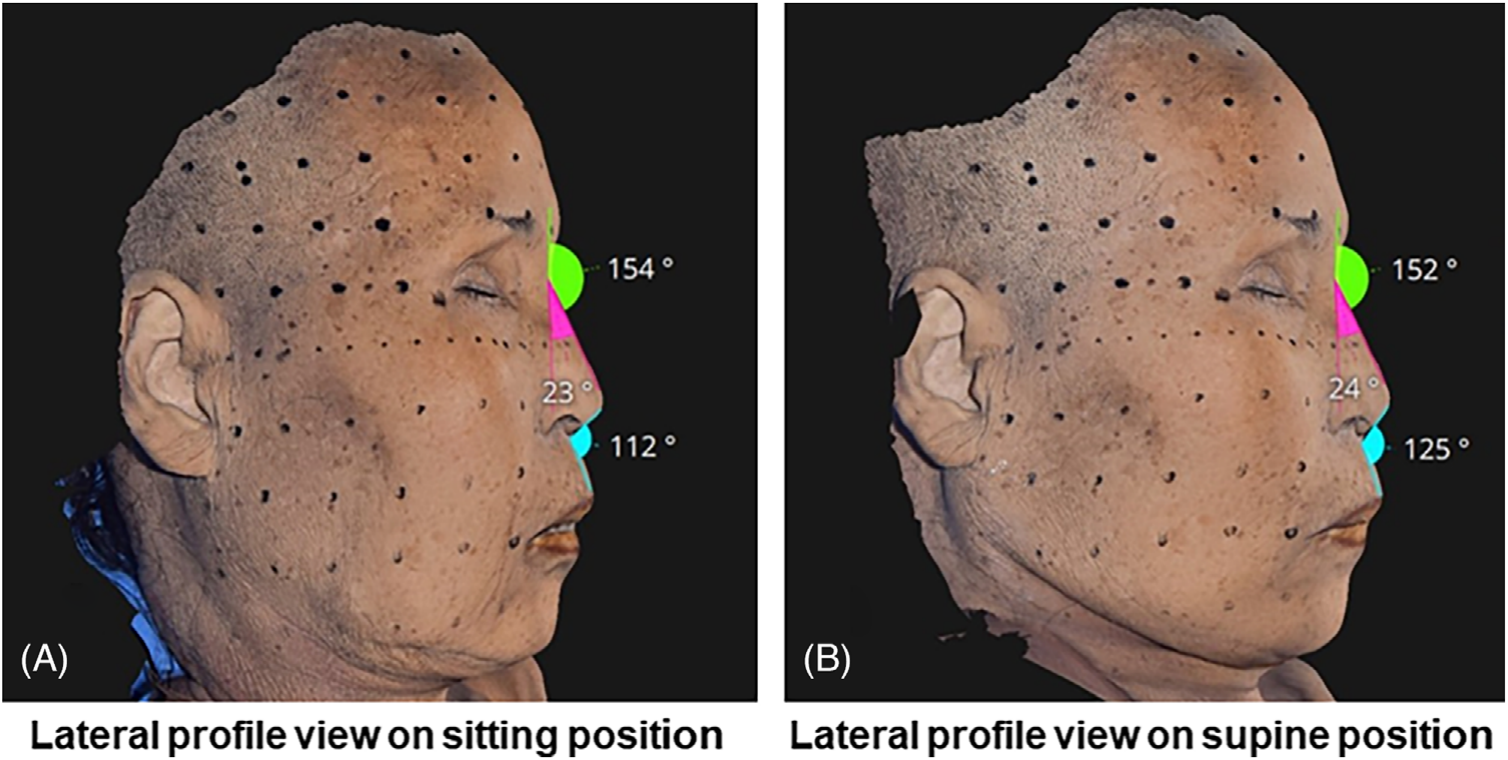
The figure illustrates alterations in facial skin and soft tissue concerning positional changes. Panel A presents a lateral profile view in a sitting position, while panel B depicts the lateral profile view in a supine position.

The study of fresh cadaveric movements of facial skin and tissues concerning postural changes reveals that transitioning from a seated to a supine position not only alters the positioning of the cadaver's head but also induces overall tension in the facial skin and soft tissues.^9^ The skin and connective tissues around the head and ears are drawn, causing a visible change in the shape and angle of the periorbital area and nasal tip. Notably, the transformation of the lower face tissues is intriguing as it demonstrates that, with postural changes, the lax skin and tissues, including those around the submental area, do not solely migrate toward the head and ears but also remain along the firm structures encompassing the chin, anterior jawline, and the angle of the mandible. This persistence contributes to the natural delineation of the chin and jawline, resulting in tauter skin. This phenomenon can be attributed to the presence of resilient structures such as the insertion of the mentalis muscle at the chin, the mandibular ligament along the boundary of the chin and jawline, and the angular tract fascia anterior to the SCM muscle within the inner angle of the mandible.^10^, ^11^ These unyielding structures play a role in the phenomenon wherein lax tissues, when shifted toward firm structures, produce a naturally effective lifting effect, aligning with the author's perspective (Figure 1).

When observing the outcomes of thread‐lifting procedures based on these principles in elderly patients, it becomes apparent that while severe skin sagging and deep wrinkles caused by aging may not be entirely resolved, there are noticeable changes in the appearance of the face. The facial structure appears less saggy, the texture of the skin and connective tissues improves, deep wrinkles become shallower, and individuals tend to look naturally younger than their actual age, acquiring a softer and more amiable facial impression.^12^, ^13^ Additionally, considering the potential exacerbation of aging‐related changes over time if thread‐lifting procedures were not undertaken, the ability of this approach to slow down facial changes associated with aging assumes considerable significance.

Moreover, in performing a natural thread‐lifting procedure that takes into account tissue movement concerning posture, an important aspect to consider is the depth at which the inserted threads reside. The preference for different techniques in thread lifting may lead some individuals to opt for the insertion of multiple relatively thin threads into the subcutaneous tissue, favoring a lighter pulling approach. Many individuals referring to this approach often mention a plane within the subcutaneous fat tissue that allows easy insertion of threads without resistance.^14^

When discussing the explanation of dual‐plane filler procedures, the author often highlights that, except for the inner aspects of the face, where vertically oriented fibrous septa firmly attach the subcutaneous fat and skin to the underlying SMAS (superficial musculoaponeurotic system) to protect the facial structure and enable facial expressions through the movement of facial muscles, there is not ample space for filler injection.^15^ This leads to the question of how such a space, suitable for performing thread lifting procedures with ease, is created during these procedures.

The reason why threads can be smoothly inserted into the subcutaneous tissue is due to the presence of spaces between the fibrous septa connecting the skin and the SMAS layer. Despite the firm attachment of these tissues through fibrous septa, the presence of these spaces allows threads to pass through. Assuming that all other conditions remain constant, the structural density difference in these fibrous septa alone leads to variations in the firmness of the subcutaneous fat tissue in upper, middle, and lower regions. If we consider fibrous septa as thin fibrous tissues, the area passing through the SMAS layer appears densely packed initially, resembling thick branches of a tree, gradually thinning out as they ascend and spreading out as they approach the dermal tissue.^16^, ^17^ To firmly anchor the skin, the fibrous tissues heading towards the skin again disperse evenly and densely, exhibiting a pattern resembling numerous closely divided thin branches. Consequently, fibrous septa passing through the SMAS layer appear robust and firm due to the dense clustering of fibrous tissues, while those situated in the middle of the subcutaneous fat layer comprise finer branches, rendering them less rigid. The retinacular cutis located closer to the dermis consists of very thin but densely distributed branches, creating a perceptibly robust structure.

**FIGURE 2:**
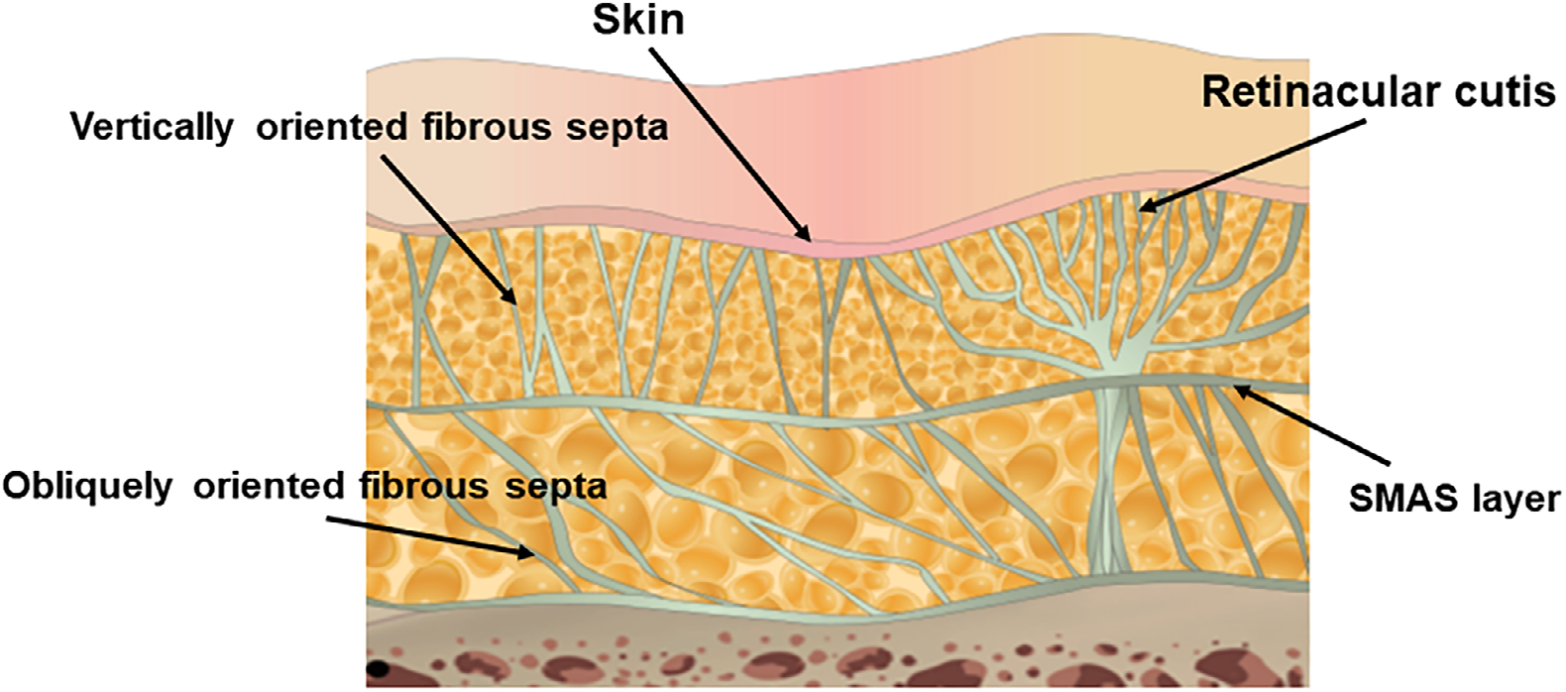
The figure demonstrates the distinction in fibrous septa between the superficial and deep fat layers.

Moreover, the density of fibrous septa within the subcutaneous tissue and the resulting strength vary across facial regions. Areas where ligaments or ligament‐like tissues hold firmly exhibit dense fibrous septa, creating a rigid sensation when threads pass through.^18^ In contrast, areas where fibers of ligamentous tissues are loosely gathered allow threads to pass more smoothly.^19^ For instance, the zygomatic ligament, regarded as one of the most robust ligaments in our face, generates substantial resistance as it traverses the lower portion of the zygomatic arch due to the density of fibrous septa within the subcutaneous tissue (Figure 2).^20^

The insertion of threads through the middle layer of subcutaneous tissue, while inducing a perception of initial skin movement due to pulling and gathering, still maintains the attachment of the skin and SMAS layer via fibrous septa. Consequently, forcefully pulling only the skin and subcutaneous tissue while the SMAS layer remains unmoved leads to a temporary sensation of tension initially, which eventually dissipates as the skin, connected to the unaltered SMAS layer, returns to its original position. Therefore, rather than excessive skin tension, a more desirable lifting effect involves a sensation of the skin layer adhering moderately toward the head and ear, ensuring sustained procedural effectiveness.

In cosmetic surgery, the reason for dissecting and pulling the SMAS layer during face‐lifting procedures ultimately stems from the difficulty in achieving prolonged lifting effects by merely dissecting and pulling the skin and subcutaneous tissues.^21^ Even skin pulled by thread insertion eventually reverts to its original position, firmly connected to the SMAS layer, and forcing excessive skin tension may engender various issues.

Nevertheless, even in recent descriptions of thread‐lifting procedures, there is a tendency to emphasize that the direction of insertion after cannula insertion should primarily target the subcutaneous tissue underneath the skin to avoid complications and hindrances. In practice, threads are inserted closer to the SMAS layer along the upper or lower surfaces of the SMAS, and even if some threads become attached to the SMAS, they are so robust that, except for regions such as the head or ear, moving the cannula does not encounter severe resistance. Upon withdrawing the inserted cannula and pulling the thread caught in the tissues, including the SMAS layer, it is observable that the skin and connective tissues, including the SMAS layer, respond well to the pull. This phenomenon is due to the deep fat layer beneath the SMAS, composed of obliquely oriented fibrous septa, unlike the superficial fat layer, allowing smoother movement of facial muscles. This area does not impede the movement of the SMAS layer due to its loose configuration and lack of firm attachment to the SMAS layer above, forming a space that allows movement, referred to as a “moving plane” beneath the SMAS due to its anatomical characteristics. Additionally, when inserting or moving the cannula, it is possible to avoid injuring important internal structures by controlling the angle of the cannula and not delving too deeply into the loose space below the SMAS layer (Figure 2).

**FIGURE 3:**
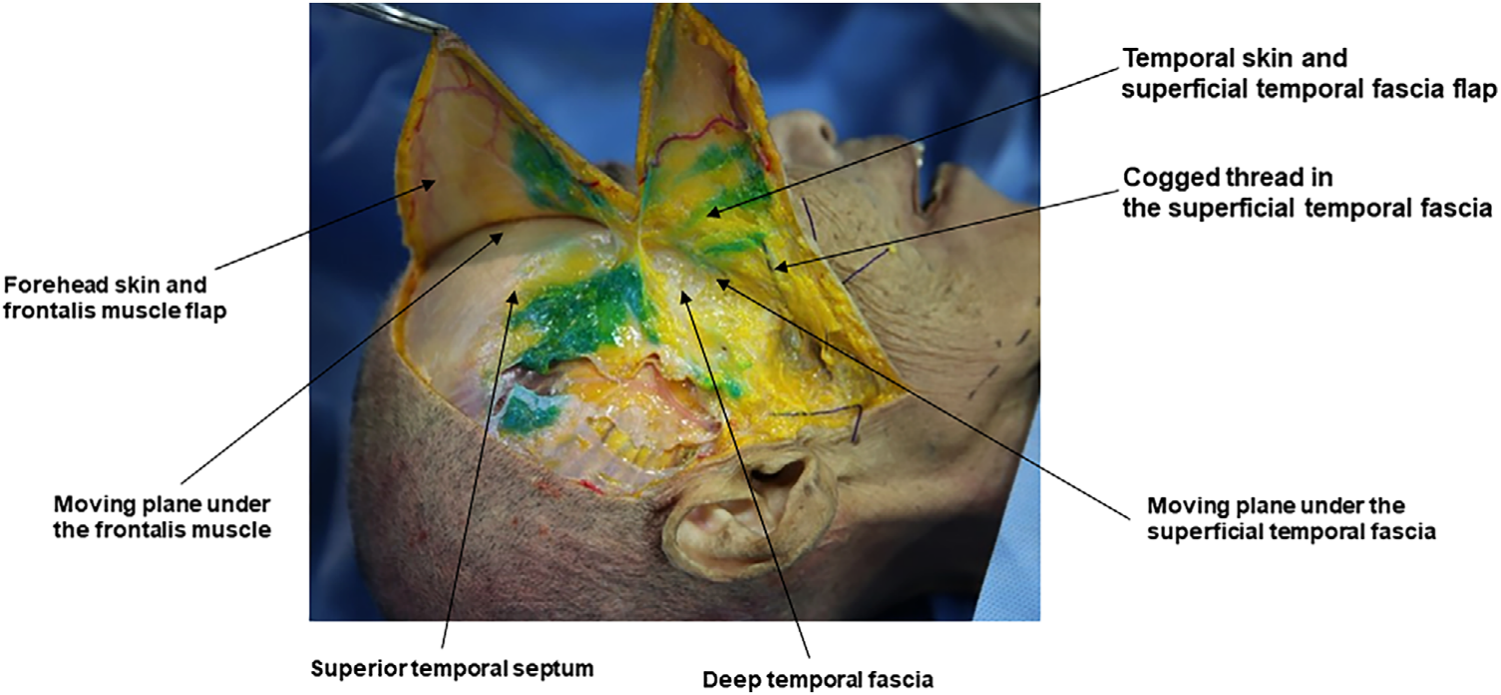
The figure delineates the shifting plane beneath the superficial musculo‐aponeurotic system (SMAS) layer for cogged thread lifting (N‐Cog, N‐finders Inc., Seoul, Korea).

Therefore, to maximize the natural repositioning effect on tissues and achieve optimal lifting and tightening outcomes during thread lifting procedures, the author suggests targeting not the skin or dermal layers but rather the SMAS layer or the surrounding tissues. By focusing on pulling these areas, the procedure aims to enhance the lifting and tightening effects through natural tissue repositioning. This approach is feasible due to the presence of a “moving plane” beneath the SMAS layer, allowing a certain degree of movement and traction of the SMAS layer, as previously discussed. The author refers to this thread‐lifting technique, which utilizes repositioning of the SMAS layer using threads, as the “SMAS Repositioning Technique with Thread Lifting.” (Figure 3).

## 3 CLINICAL APPLICATION

**FIGURE 4:**
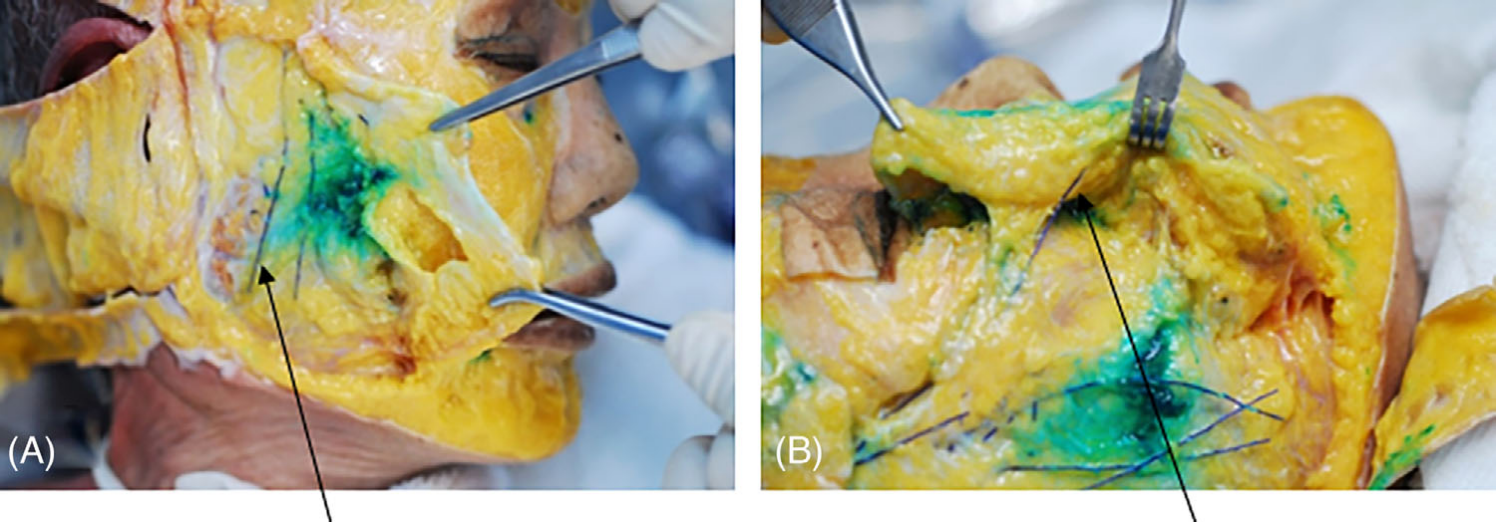
An approach entails placing the patient on a tilting table oriented against gravity, facilitating the concentration of the superficial musculo‐aponeurotic system (SMAS) and reticular cutis toward the temple prior to thread insertion. Panel A demonstrates the face's inclination in the antigravity orientation, while panel B showcases the consolidation of the reticular cutis and SMAS (N‐Fix, N‐finders Inc., Seoul, Korea).

There are generally two approaches to this procedure. One involves positioning the patient on a tilting table in the antigravity direction, allowing the SMAS and reticular cutis to be densely located toward the temple before inserting the threads (refer to Figure 4 and Video S1). In the video demonstration, N‐Finders product called N‐Cog and N‐Fix, which are the main thread of this SMAS repositioning concept. This method offers the advantage of pre‐assessing the effects of thread insertion as the shape is already established, thereby reducing the risk of developing dimples. Additionally, it minimizes the likelihood of adverse effects such as bleeding or nerve paralysis by avoiding excessive pulling on nerves or blood vessels.

**FIGURE 5:**
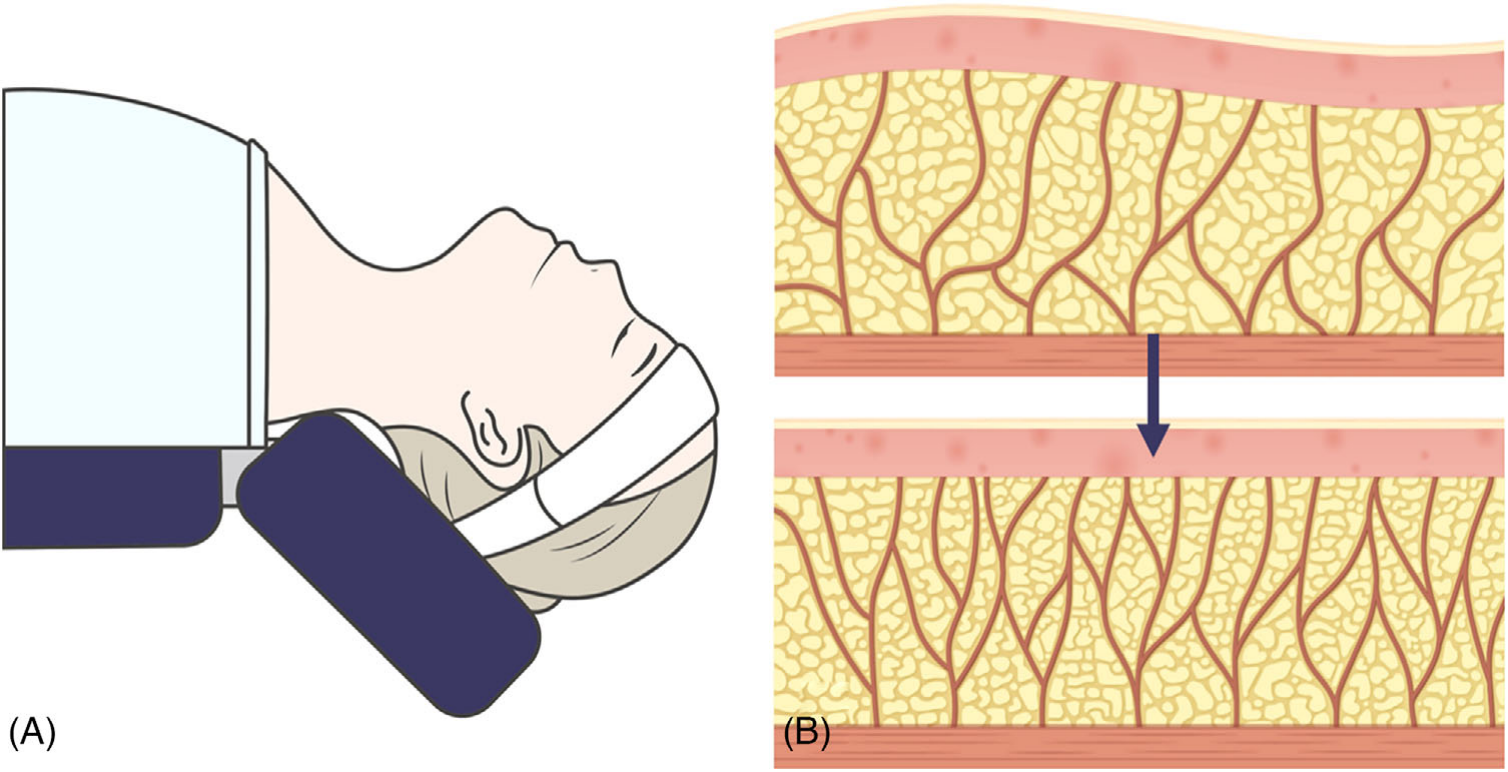
In addition to the method utilizing gravity, another approach involves manually pushing the tissue towards the temple on the skin surface using the palm of the hand, creating a dense positioning of the reticular cutis towards the temple before inserting the threads.

**FIGURE 6:**
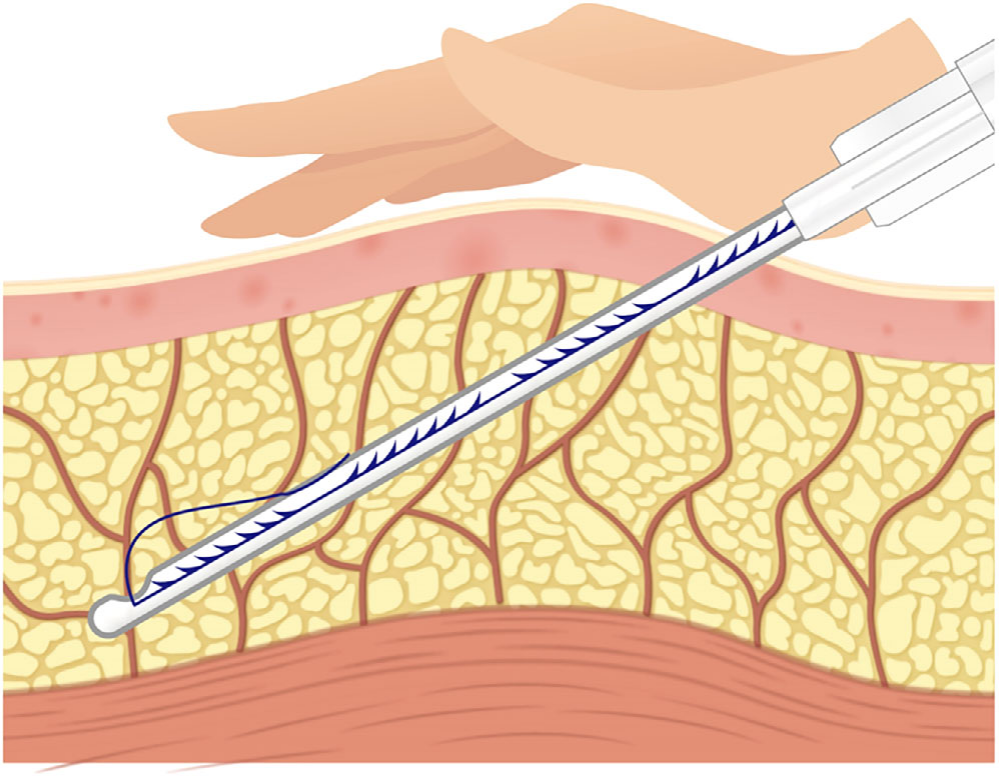
Ensuring a compact alignment of the superficial musculo‐aponeurotic system (SMAS) and reticular cutis before inserting the threads enables them to sustain this configuration while eliciting a lifting effect (N‐Cog, N‐finders Inc., Seoul, Korea).

Another approach would be, in the absence of a tilting table utilizing gravity, an alternative method involves manually pushing the tissue toward the temple on the skin surface using the palm of the hand before inserting the threads (Figure 5). It is advisable to confirm the absence of nerve compression or other issues by applying pressure with the palm for approximately one minute before thread insertion. By establishing a dense position of the SMAS and reticular cutis before thread insertion, the threads can maintain this shape while producing a lifting effect (Figure 6).

## 4 DISCUSSION

**FIGURE 7:**
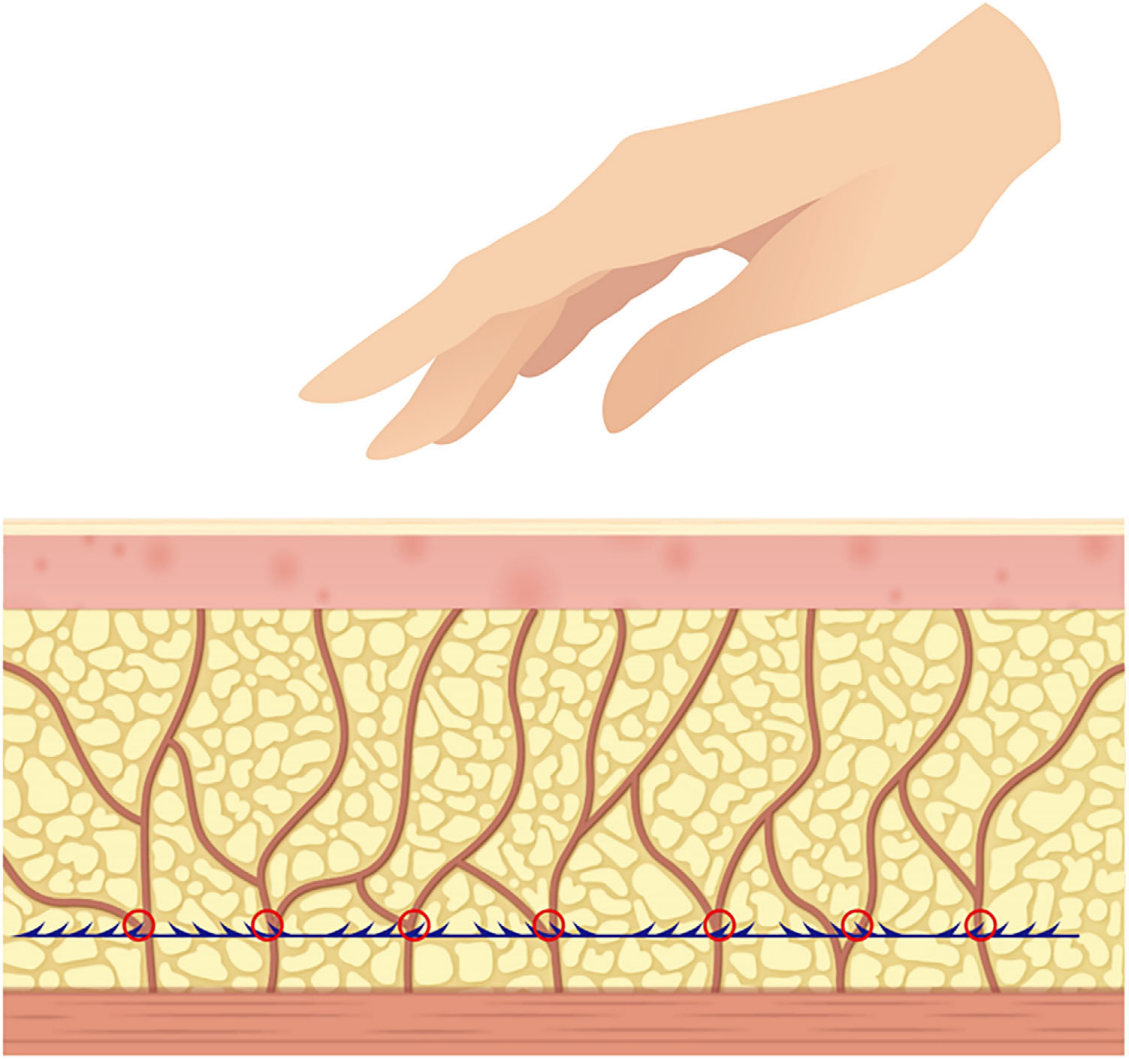
The figure showcases cogged threads inserted along the supra SMAS plane (N‐Cog, N‐finders Inc., Seoul, Korea).

In fresh cadaver studies conducted for thread lifting procedures, observations reveal that, without intentionally inserting threads close to the skin, practitioners aim to mimic the depth of insertion in actual patients. Delicately inserting the cannula to the same depth as during procedures and subsequently placing threads, upon dissecting the skin, shows that in a significant number of cases, threads are found to traverse along or partially penetrate the SMAS layer. In instances where the skin and subcutaneous tissues are thin, it is observed that threads are predominantly inserted along the underside of the SMAS layer. (Figure 7).

The possibility of threads penetrating the SMAS layer itself stems from the structural composition of the SMAS when examined in detail. Rather than being a single thick layer of muscle, the SMAS consists of a superficial fascia comprising three layers.^22^ Within this structure, the SMAS is not merely a thick muscular layer; instead, it consists of a thin and resilient membranous layer sandwiched between upper and lower fatty layers, forming a superficial fascia (Figure 2).

As mentioned earlier, the skin and SMAS layer are firmly connected through vertically oriented fibrous septa and retinacular cutis. Therefore, when threads pass through the SMAS or its surrounding areas, and by pulling the SMAS layer around the moving plane beneath the SMAS, a natural lifting effect is achieved. Consequently, the movement of the skin and subcutaneous tissues occurs in accordance with this relocation, demonstrating the lifting effect. This understanding involves the concept that when the SMAS layer is relocated, the skin and subcutaneous tissues connected to the SMAS naturally move alongside the periSMAS tissue. Hence, there is a shift toward performing thread‐lifting procedures even for individuals of relatively advanced age, emphasizing the changing approach toward implementing thread‐lifting procedures for older individuals nowadays.

Clinically observed phenomena indicate that when the nodules of the thread impact the dermal tissues beneath the skin surface, it often results in the appearance of dimples or irregularities on the skin. Conversely, if the nodules of the thread catch and pull within the deep facial muscle layer, it may lead to awkward facial expressions and cause discomfort or pain when moving the face. During actual procedures, when passing through the fat compartments just above or below the SMAS layer, practitioners often experience a smooth movement of the needle or cannula without resistance. However, inserting the needle or cannula into the deeper fascial layers of the facial muscles results in less movement upon manipulation and leads to patient discomfort or pain.

**FIGURE 8:**
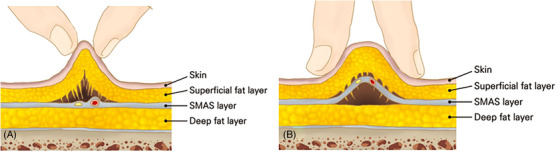
The figure illustrates the technique of shallow pinch for subcutaneous tissue traction (panel A) and deep pinch for superficial musculo‐aponeurotic system (SMAS) layer traction (panel B).

When a needle or cannula is properly inserted just above or below the SMAS layer, or within the SMAS layer, raising the needle or cannula reveals that the layer of skin and subcutaneous tissue above it lifts uniformly to a certain thickness equal to the length of the needle or cannula. To determine the appropriate depth for the procedure, a useful method involves utilizing the pinch technique, where the practitioner uses their non‐dominant hand (usually the left hand) to grasp and pull the tissue to measure its thickness. Studies employing the pinch technique generally demonstrate that intentionally thinning and elevating the skin tissue with the tips of the thumb and index finger often results in mainly lifting the subcutaneous tissues above the SMAS layer, with minimal disturbance to the SMAS layer itself, which is barely touched on the surface (Figure 8) When using the broader and thicker part of the finger pad, rather than just the tips of the thumb and index finger, to grasp and lift the tissue in the outer cheek area, it can lead to a somewhat rounded elevation of the skin and subcutaneous tissues, including the SMAS layer.

**FIGURE 9:**
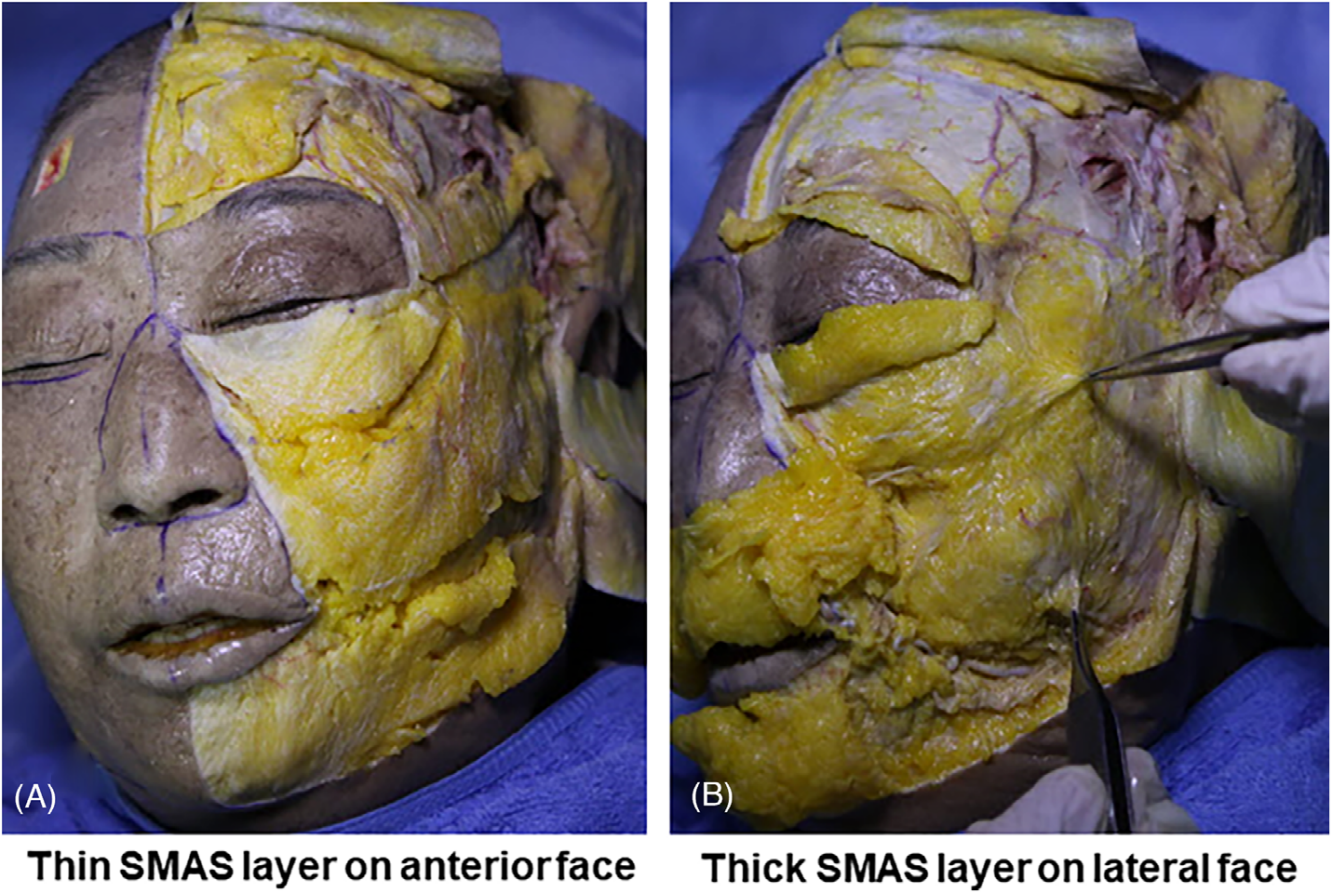
The figure displays variations in superficial musculo‐aponeurotic system (SMAS) thickness and tightness concerning anteior face (A) and lateral face (B).

It is crucial to note that on the lateral side of the face near the ear, the SMAS layer is thicker and firmer compared to the inner aspects of the face. When lifting tissue in this area, despite grabbing and elevating the tissue firmly, the SMAS layer may not elevate significantly above, but rather the tissue being held might only slightly lift just beneath the subcutaneous tissue (Figure 9). Therefore, when inserting needles or cannulas for thread lifting, as long as the tissue is not pushed too deeply beneath the grasped tissues, the probability of the thread going deep under the SMAS layer is low.

However, in the inner aspects of the face, the SMAS layer is thinner, and the deep fat layer is thicker. Carelessly grasping and pinching too thickly in this area can cause the SMAS layer and even the deep fat layer underneath to be inadvertently lifted together. This could result in the thread being positioned deeper than intended, possibly perforating the mucous membrane inside the mouth. Moreover, if the thread grasps the tissue too thickly, the ultimate efficacy of the procedure might be compromised. Therefore, caution is strongly advised to prevent these potential complications.

The early stages of thread lifting involved the belief that maximum pulling and tensioning of the skin and subcutaneous tissues using thread cones were necessary to maintain some residual effect even as the threads naturally loosened over time. However, with improvements in thread shapes and qualities for lifting and the enhancement of lifting materials, there's no longer a strong necessity to excessively tension tissues during the initial stages. It is now recognized that the contemporary concept of thread lifting focuses less on the forced lifting of superficial skin and subcutaneous tissue but rather on the natural repositioning of deeper tissue layers such as the SMAS and its surrounding tissues. After tissue repositioning, it is crucial to stabilize the relocated tissues to prevent them from moving back to their original positions. Hence, the tension applied by the threads, capable of withstanding the direction of gravity and maintaining the tissues in their adjusted position, becomes vital.

In the past, U‐type long threads were frequently used for this purpose, but nowadays, there's a wide variety of PDO threads available in different thicknesses and shapes. Advancements in manufacturing techniques and procedural instruments have facilitated the use of convenient I‐type threads for various enhancements beyond lifting the outer jawline, including addressing issues like eyebrow drooping, eye hollows, forehead lines, marionette lines, and neck sagging.

The choice of threads depends on factors such as the thickness and weight of the patient's skin and tissues, the extent of sagging, depth of wrinkles, and the patient's preferences. In some cases, a combination of multiple threads may be suitable. When employing SMAS repositioning‐based thread lifting, an essential consideration is determining the direction in which the tissues are stretched, guiding the insertion direction of the threads accordingly. Once the thread insertion direction is established, decisions need to be made regarding the entry point of the threads, thread thickness, quantity, insertion depth, and awareness of important anatomical structures and tissues prone to thread entanglement. These considerations are fundamental regardless of the facial area targeted for thread lifting.

## CONFLICT OF INTEREST STATEMENT

I acknowledge that I have considered the conflict of interest statement included in the “Author Guidelines.” I hereby certify that, to the best of my knowledge, no aspect of my current personal or professional situation might reasonably be expected to significantly affect my views on the subject I am presenting.

## STATEMENT OF HUMAN AND ANIMAL RIGHTS, OR ETHICAL APPROVAL

This article does not contain any studies with human participants or animals performed by any of the authors.

## Supporting information



Supporting Information

## Data Availability

Data sharing is not applicable to this article as no new data were created or analyzed in this study.
